# Risk factors of electrical status epilepticus during sleep in children with benign childhood epilepsy with centro-temporal spikes

**DOI:** 10.12669/pjms.40.4.7674

**Published:** 2024

**Authors:** Xiufeng Wang, Yanling Zhang, Ruixue Sun, Na Kong

**Affiliations:** 1 Xiufeng Wang, Department of Pediatrics, Xingtai People’s Hospital, Xingtai 054000, Hebei, P.R. China; 2 Yanling Zhang, Department of Pediatrics, Xingtai People’s Hospital, Xingtai 054000, Hebei, P.R. China; 3 Ruixue Sun, Department of Pediatrics, Xingtai People’s Hospital, Xingtai 054000, Hebei, P.R. China; 4 Na Kong, Department of Pediatrics, Xingtai People’s Hospital, Xingtai 054000, Hebei, P.R. China

**Keywords:** Benign childhood epilepsy with centrotemporal spikes, Electrical status epilepticus during sleep, Risk factor

## Abstract

**Objective:**

To explore risk factors of electrical status epilepticus during sleep in children with benign childhood epilepsy with centro-temporal spikes (BECT).

**Methods:**

This is a clinical comparative study. The subjects of study were 67 children with BECT from the Outpatient Department of Pediatric Neurology in Xingtai People’s Hospital from January 2019 to January 2022. According to the occurrence of ESES, the enrolled children were divided into control group which included BECT children without ESES and the observation group which included BECT children with ESES. Compared differences of the two groups in the age of first seizure, the frequency of seizures before treatment, the classification of treatment drugs, cranial MRI, and discharge side of electroencephalogram (EEG).

**Results:**

There was no statistical difference between the two groups in the frequency of seizures before treatment, the classification of treatment drugs, cranial MRI, and the distribution of EEG discharges in the left and right cerebral areas(*P*>0.05). Statistical differences were observed in the age of the first seizure, whether the seizures occurred after treatment, and EEG discharges in unilateral/bilateral cerebral areas (*P*<0.05). Furthermore, the collinearity test and Logistic regression analysis showed that the age of the first seizure, the frequency of seizures before treatment, and whether the seizures occurred after treatment were independent risk factors for the occurrence of ESES in BECT (*P*<0.05).

**Conclusion:**

Clinically, the occurrence of ESES in children with BECT may be related to the younger age of the first seizure, higher frequency of seizures before treatment, and the occurrence of seizures after treatment.

## INTRODUCTION

Benign childhood epilepsy with centrotemporal spikes (BECT) is a common benign and focal epilepsy in the pediatric age group, most of which disappear naturally in adolescence, without affecting intelligence and cognition of the affected children. In the past, it was thought that the prognosis of BECT was favorable, which, however, was not the case according to a growing body of research.^1^ There is a significantly increased discharge during non-rapid eye movement (NREM) sleep in some children, with a discharge index of > 85%, which is called electrical status epilepticus during sleep (ESES).^2^-^3^ ESES is generally characterized by diverse forms of seizures and poor response to drugs. Such patients often have increased frequency of seizures and/or difficulty in seizure control, as well as neuropsychological function damage to varying degrees.

It may result in cognitive regression, psychobehavioral abnormalities and neuropsychological function damage in children to some extent, which may affect the long-term prognosis consequently.^4^,^5^ Therefore, efforts should be made to suppress the presence of ESES for BECT patients with ESES. However, it is still unclear with respect to the causes of ESES in BECT at present. Accordingly, by collecting relevant clinical data of BECT cases, the present study was performed to evaluate the influencing factors of ESES in BECT through multivariate Logistic regression analysis, to explore potential solutions to reduce the mental impairment of children caused by ESES, so as to benefit early diagnosis and individualized intervention of such children.

## METHODS

This is a clinical comparative study. The subjects of study were 67 children with BECT from the Outpatient Department of Pediatric Neurology in Xingtai People’s Hospital from January 2019 to January 2022. All children were enrolled according to the inclusion and exclusion criteria with complete clinical data. There were 39(58.21%) males and 28 (41.79%) females, with the age of first seizure of 3-15 years old and an average age of (6.90 ± 2.36) years.

### Ethical Approval

This study was approved by the Ethics Committee of Xingtai People’s Hospital (2020[039]; April 16, 2020), and written informed consent was obtained from all patients’ guardians.

### Inclusion criteria:

- Children with typical central-temporal spikes indicated by video electroencephalogram (V-EEG) monitoring, showing typical characteristics of BECT in terms of duration and frequency, and meeting the diagnostic criteria of BECT in the classification of epilepsy syndromes proposed by International League Against Epilepsy (ILAE) in 2001, simultaneously, children who met the diagnostic criteria of ESES: the diffuse or nearly continuous Spike-Wave (SW) discharges during NREM sleep, accounting for 50% of the period of NREM sleep.^6^
- Children without abnormality in imaging examination (cranial magnetic resonance imaging (MRI)/CT) or neurophysical examination.

### Exclusion criteria:

- Children with organic lesions indicated by cranial MRI/CT.
- Children with other nervous system diseases such as cerebral palsy or developmental disability intellectual.
- Children suffering from other related epilepsy syndromes with ESES.
- Children with incomplete clinical data who did not have regular follow-up and re-examination of EEG.

General data of the included children were collected, including name, age of first seizure, gender, family history, the frequency of seizures before treatment, form of onset, treatment drugs, birth, blood biochemical indicators, intelligence, cranial MRI, the frequency of EEG discharges, location of EEG discharges, etc.

According to the occurrence of ESES, the enrolled children were divided into two group, of which the control group included BECT children without ESES, and the observation group included BECT children with ESES.

The intelligence of children was evaluated by the modified Wechsler Intelligence Scale for Children-Third Edition (WISC-R)^6^ in China. The scale included 12 tests of vocabulary, memorization, common sense, understanding, similarity, arithmetic, arrangement, mapping, jigsaw puzzle, building block, maze and decoding. The full intelligence quotient (FIQ) grading criteria were described as follows: normal: >90 points; below normal: 80~89 points, critical: 70~79 points; and mental retardation:<70 points.

The digital long range video electroencephalograph (NIHON KOHDEN CORP, V-1200) was applied to monitor children’s video electroencephalogram during waking and sleeping periods. The 19 disc-shaped scalp electrodes were placed over the scalp according to an international convention (10-20 System), with the ear clip electrode as the reference electrode. The monitoring results were recorded and analyzed in unipolar and bipolar leads by Neuroelectrophysiological professionals. The waking period included quiet state, hyperventilation and eyes-opening and -closing reaction; and the sleeping period included at least one sleep cycle. The monitoring time lasted for 4-5 hour: (1) deterioration in EEG: (a) during waking period: SW (2-3Hz) discharge widely in various regions; (b) during NREM sleep: discharge index > 50%; (c) during waking and sleeping periods: significantly increased discharge, with spike and SW discharges of extremely high voltage appeared in the middle temporal area and central area; (2) ESES: Spike-Wave Indices (SWI) ≥ 50%.

### Statistical analysis

SPSS 26.0 software was used for statistical analysis. The quantitative data conforming to normal distribution were described by mean±standard deviation, and those not conforming to normal distribution were expressed by median and quartile. Group t-test was used for inter-group comparison of quantitative data. The qualitative data were presented by rate, and compared by chi-square test. The collinearity of each factor was determined through VIF and tolerance in regression analysis. Meanwhile, Logistic regression analysis was performed to explore factors affecting the occurrence of ESES in BECT. *P*<0.05 indicated that the difference was statistically significant.

## RESULTS

There was no statistical difference between the two groups in the frequency of seizures before treatment, the classification of treatment drugs, past history, cranial MRI, and the distribution of EEG discharges in the left and right cerebral areas (*P*> 0.05). While statistical differences were observed in the age of the first seizure, whether the seizures occurred after treatment, and EEG discharges in unilateral/bilateral cerebral areas (*P* < 0.05).

Based on clinical experience, the variables with *P*<0.2 when comparing clinical data between groups were selected for regression analysis. Through collinearity diagnosis, it was found that the VIF value for the collinearity statistics for variables affecting the occurrence of ESES in BECT was <5, and the tolerance value was >0.1 significantly. Meanwhile, the conditional index of dimension 5 was ≥10, but the variance proportion of closing to 1.0 was found in the age of first seizure only. Collectively, these data revealed a weak degree of collinearity of each factor, suggesting further multivariate regression analysis, as shown in Tables-II and III.

**Table-I:** Comparison of clinical data between the two groups [±*s*, n(%)].

Category	BECT (n = 54)	BECT+ESES (n = 13)	t/c^2^	P
The age of first seizure	7.41 ± 2.32	4.77 ± 0.83	4.02	<0.001
The frequency of seizures before treatment	3.56 ± 1.85	4.77 ± 2.65	1.94	0.056
The classification of treatment drugs			5.08	0.079
LEV	24 (44.44)	8 (61.54)		
OXC	16 (29.63)	0 (0.00)		
Others	14 (25.93)	5 (38.46)		
Whether the seizures occurred after treatment				<0.001*
With	8 (14.81)	13 (100.00)		
Without	46 (85.19)	0 (0.00)		
Past history			0.84	0.358
With febrile convulsion	7 (12.96)	3 (23.08)		
Without febrile convulsion	47 (87.04)	10 (76.92)		
Cranial MRI			2.75	0.097
With abnormality	4 (7.40)	3 (23.10)		
Without abnormality	50 (92.60)	10 (76.90)		
Distribution of EEG discharges in unilateral/bilateral cerebral areas			4.94	0.026
Unilateral	31 (57.41)	3 (23.07)		
Bilateral	23 (42.59)	10 (76.93)		
Left and right sides of EEG			4.94	0.084
Left	11 (20.37)	1 (7.69)		
Right	20 (37.04)	2 (15.38)		
Others	23 (42.59)	10 (76.92)		

***Note:***

* Comparison using Fisher’s exact probability test.

**Table-II:** The collinearity statistics of factors affecting the occurrence of ESES in BECT

Category	Tolerance	VIF
The age of first seizure	0.971	1.030
The frequency of seizures before treatment	0.409	2.447
Whether the seizures occurred after treatment	0.643	1.555
EEG discharges in unilateral/bilateral cerebral areas	0.462	2.164

**Table-III:** The collinearity diagnosis of factors affecting the occurrence of ESES in BECT.

Variance proportion						

Dimension	Characteristic value	Conditional indicator	The age of first seizure	Whether the seizures occurred after treatment	EEG discharges in unilateral/bilateral cerebral areas	The frequency of seizures before treatment
1	3.95	1.00	0.01	0.02	0.01	0.01
2	0.64	2.48	0.04	0.35	0.03	0.00
3	0.29	3.72	0.02	0.51	0.47	0.01
4	0.08	7.22	0.26	0.13	0.43	0.78
5	0.05	9.40	0.68	0.00	0.06	0.21

The multivariate Logistic analysis (regression method: Enter) was performed by using the indicators with differences between groups as independent variables (categorical variable assignment: whether the seizures occurred after treatment: 0= no, 1= yes; EEG discharges in unilateral/bilateral cerebral areas: 0= unilateral, 1= bilateral, with assignment =1 as the reference), and the occurrence of ESES in BECT as the dependent variable. Corresponding results showed that the age of first seizure, the frequency of seizures before treatment, and whether the seizures occurred after treatment were independent risk factors for the occurrence of ESES in BECT (*P*< 0.05; Table-IV).

**Table-IV:** Univariate and multivariate regression analysis of influencing factors for the occurrence of ESES in BECT.

Category	β value	Standard error	95%CI	P
The age of first seizure	-0.329	0.012	-0.080~-0.031	0.000
The frequency of seizures before treatment	-0.277	0.022	-0.097~-0.010	0.016
Whether the seizures occurred after treatment	0.806	0.076	0.535~0.838	0.000
EEG discharges in unilateral/bilateral cerebral areas	0.061	0.083	-0.118~0.215	0.561

## DISCUSSION

In the comparison of the two groups of children with BECT, there was no statistical difference between the two groups in the frequency of seizures before treatment, the classification of treatment drugs, past history, cranial MRI, and the distribution of EEG discharges in the left and right cerebral areas (*P*> 0.05). While statistical differences were observed in the age of the first seizure, whether the seizures occurred after treatment, and EEG discharges in unilateral/bilateral cerebral areas (*P*< 0.05). In general, the discharges level is expressed by SWI during slow-wave sleep, and SWI ≥ 85% is one of the recognized criteria for diagnosing ESES.

Afterwards, some scholars also put forward different criteria for ESES (e.g., SWI of 90%, 85%, 60%, 50% and 15%).^7^-^10^ Without specification of the threshold, ILAE only requires that the SW discharges must have the characteristics of “continuous” and “diffuse”. In this study, whether SWI was >50% was used as the diagnostic criterion of ESES. Clinically, drug treatment for epilepsy mainly includes antiepileptic drugs (AEDs), hormones, etc.^11^, among which levetiracetam (LEV) and oxcarbazepine (OXC) are the first-line drugs for treatment. Specifically, LEV is a relatively new second-generation AEDs, and it can bind selectively to proteins related to neurotransmitter release, which can work rapidly.^12^

Moreover, OXC is a new-generation AEDs derived from traditional AEDs, with few side effects. It plays an anti-convulsive role mainly by blocking sodium channel and promoting potassium conduction, and has a good therapeutic effect on children.^13^ In this study, all the enrolled children received monotherapy basically, and there was no significant difference in the classification of treatment drugs (*P*> 0.05).

In an overseas research carried out by Kessi et al.^14^, the proportion of patients with good cognitive ability was higher in patients receiving monotherapy than in those receiving dual-drug treatment or multi-drug treatment. However, by comparing the efficacy of the two groups after treatment (i.e., whether the seizure occurred again after treatment), the re-seizure rate of patients without ESES was significantly lower than that of patients with ESES (*P*< 0.05). Most children showed no seizures after drug treatment, and the effective rate was 68.66% (46/67). Nevertheless, for children with ESES, both traditional and new-generation AEDs were ineffective due to individual differences, which was similar to that reported by Fu JX et al.^15^

Furthermore, considering our clinical experience and differences between the two groups, Logistic analysis was performed by including the frequency of seizures before treatment, the age of first seizure, and whether the seizures occurred after treatment. The results showed that younger age of first seizure, higher frequency of seizures before treatment, and the occurrence of seizures after treatment were independent risk factors for the occurrence of ESES in children with BECT. Similarly, a domestic research conducted by Zhou LP and Malik MA et al.^16^,^17^ revealed seizures after initial treatment in patients with BECT, which was a high-risk factor for the occurrence of ESES. Meanwhile, Sun et al.^18^ also proposed that the electroencephalogram of drug-resistant epilepsy also had the characteristics of extensive multiple spikes.

Moreover, as reported by Roebber et al.^19^, there was a certain relationship between spike-wave discharge during sleep and the cortico-thalamic circuit. In their research, it was found that during non-REM sleep, the amplitude of the cortico-thalamic circuit led to the appearance of sleep spindles and K-complexes, promoting the occurrence of ESES; while after the administration of AEDs, patients might showed increased power of sleep spindles and reduced amplitude of slow waves, exhibiting decreased coupling strength of spindles to slow waves, which would produce an impact on both the coupling phase and coupling strength; all these factors also affected sleep and cognitive ability of the patients, suggesting that there might be no improvement in spindle formation or consolidation of the connection between the hippocampus-cortex-thalamus circuit related to sleep after treatment with major second-generation AEDs (e.g., LEV and OXC) in children with ESES. With the increase of age in children with ESES, the specific EEG pattern during slow wave sleep may be disappeared at the age of 11-14 years, suggesting that its occurrence and development are dependent on age.^20^ Similar to this study, He W et al.^21^ indicated that BECT children with age of onset and duration of epilepsy had higher risk of developing ESES, which might affect neuropsychological function and cognitive level of these children.

### Limitations of the study

The conclusions is still controversial due to the small sample size and different criteria used by the current studies. Simultaneously, in view of the research design of single-center study with smaller sample size, multi-center studies are needed based on larger sample size for further verification.

## CONCLUSIONS

This study shows that BECT children with younger age of the first seizure, higher frequency of seizures before treatment, the occurrence of seizures after treatment has higher risk of developing ESES during the development of the disease. Findings in our study suggest that during the diagnosis and treatment of BECT, attention should be paid to children with the above characteristics. It is recommended to increase the frequency of monitoring via EEG, and keep vigilant against the occurrence of ESES, so as to avoid the cognitive impairment of children.

### Authors’ Contributions:

**XW:** Carried out the study, collection of data, drafted the manuscript, are responsible and accountable for the accuracy and integrity of the work.

**YZ** and **RS:** Performed the statistical analysis and participated in its design.

**NK:** Participated in acquisition, analysis, or interpretation of data and drafting the manuscript.

All authors have read and approved the final manuscript.
